# Diversity of Pneumolysin and Pneumococcal Histidine Triad Protein D of *Streptococcus pneumoniae* Isolated from Invasive Diseases in Korean Children

**DOI:** 10.1371/journal.pone.0134055

**Published:** 2015-08-07

**Authors:** Ki Wook Yun, Hyunju Lee, Eun Hwa Choi, Hoan Jong Lee

**Affiliations:** 1 Department of Pediatrics, Seoul National University College of Medicine, Seoul, South Korea; 2 Department of Pediatrics, Seoul National University Children’s Hospital, Seoul, South Korea; 3 Department of Pediatrics, Seoul National University Bundang Hospital, Seongnam, South Korea; Instituto Butantan, BRAZIL

## Abstract

Pneumolysin (Ply) and pneumococcal histidine triad protein D (PhtD) are candidate proteins for a next-generation pneumococcal vaccine. We aimed to analyze the genetic diversity and antigenic heterogeneity of Ply and PhtD for 173 pneumococci isolated from invasive diseases in Korean children. Allele was designated based on the variation of amino acid sequence. Antigenicity was predicted by the amino acid hydrophobicity of the region. There were seven and 39 allele types for the *ply* and *phtD* genes, respectively. The nucleotide sequence identity was 97.2%-99.9% for *ply* and 91.4%-98.0% for *phtD* gene. Only minor variations in hydrophobicity were noted among the antigenicity plots of Ply and PhtD. Overall, the allele types of the *ply* and *phtD* genes were remarkably homogeneous, and the antigenic diversity of the corresponding proteins was very limited. The Ply and PhtD could be useful antigens for universal pneumococcal vaccines.

## Introduction

*Streptococcus pneumoniae* is one of the most important pathogens responsible for otitis, sinusitis, and pneumonia and is a predominant cause of meningitis and bacteremia [1]. Since the introduction of the 7-valent pneumococcal conjugate vaccine (PCV7), a decrease in the incidence of invasive pneumococcal disease (IPD) caused by vaccine serotypes has been observed in pediatric and non-pediatric populations [2, 3]. Currently, licensing of the 10- and 13-valent PCVs heralds a new era in the control of pneumococcal diseases.

However, the licensed PCVs elicit protective antibodies against only the serotypes included in the vaccine formulation. Furthermore, an increase of non-PCV7 serotypes in IPD has been observed in many countries [1]. To broaden the protection afforded by the vaccine, therefore, it has been suggested that a serotype-independent vaccine be developed, and virulence protein components have been proposed as vaccine candidates [4]. Various virulence proteins of *S*. *pneumoniae* have been investigated as candidate antigens for protein-based vaccines [5]. These protein antigens are expected to be immunogenic in young children without requiring chemical conjugates or other carriers. Among them, the immunogenicity and prevalence of pneumolysin (Ply) and pneumococcal histidine triad protein D (PhtD) make these proteins the most promising vaccine candidates for preventing pneumococcal disease [5–7].

Ply, a 53-kDa cytoplasmic cholesterol-dependent pore-forming toxin, is thought to be an important virulence factor that exerts lytic effects on many cell types. In addition to mediating cell lysis, sub-lytic levels of Ply have a number of effects on host systems, including complement activation and the induction of proinflammatory mediators [8]. Recently, Shak et al. reported a novel role for Ply in the assembly of *S*. *pneumoniae* biofilms [9]. PhtD is a relatively large surface protein of 110 kDa and is thought to be involved in multiple functions, including metal ion homeostasis, evasion of complement deposition, and adherence of bacteria to host cells [7]. The protective efficacy of immunization with a Ply toxoid and PhtD has been demonstrated in animal models [10–13] and clinical trials [14–16].

Variation in the amino acid sequence of a candidate protein may influence the immunogenicity of a vaccine based on the presence of a single allele of given protein [17]. Although the antigenicity of a candidate protein may appear to be good, a protein with high sequence diversity does not make an ideal candidate vaccine. Few studies have investigated the sequence conservation of the *ply* and *phtD* genes among several pneumococcal serotypes of invasive isolates. Thus, we aimed to characterize the genetic diversity and antigenicity of the two most promising antigens, Ply and PhtD, for a protein vaccine.

## Materials and Methods

This study was approved by the Institutional Review Board of Seoul National University Hospital (IRB registration number 1306-071-527). The Ethics Committee allowed a waiver of informed consent because this study included only the information of bacteria without any information of human from whom the bacteria was obtained.

### Strains

A total of 173 invasive pneumococcal isolates were obtained from children <18 years of age at the Seoul National University Children’s Hospital between 1991 and 2011. An ‘invasive isolate’ was defined as the isolate obtained from a normally sterile body fluid, such as blood, cerebrospinal fluid, pleural fluid, ascites, or joint fluid. Each isolate was identified using standard microbiological techniques, including observations of colony morphology, hemolysis pattern, and optochin susceptibility tests. Isolates were kept at -80°C until use. Serotype was determined using the Quellung reaction and by polymerase chain reaction (PCR) followed by sequencing of capsular genes [18].

### Sequence analysis of the *ply* and *phtD* genes

Extraction and purification of DNA from pneumococcal colonies were performed using a QIAamp Kit (QIAGEN GmbH, Hilden, Germany) according to the manufacturer’s protocol. Sequence analyses of the *ply* and *phtD* genes were performed for all 173 invasive pneumococcal isolates. The complete sequences of the *ply* and *phtD* genes were amplified with newly designed primers and sequenced using conditions as described previously [19]. All primers used in this study are listed in Table 1. All sequences generated in this study have been deposited in GenBank under accession numbers KP110598 to KP110770 for the *ply* gene and KP127680 to KP127852 for the *phtD* gene.

**Table 1:** Oligonucleotide primers used for polymerase chain reaction and sequencing in this study.

Target gene	Primer name	Sequence (5’ → 3’)	Reference
***Ply***	Ply_9Y	CGGGATCCGGCAAATAAAGCAGTAAATGACTTT	19
	Ply_9Z	GACGGAGCTCGACTAGTCATTTTCTACCTTATC	
	Ply_4V	CAATACAGAAGTGAAGGCGG	
***PhtD***	PhtD_F2	GACCCACAAAATGACAAGACC	This study
	PhtD_F3	TCGTTATATCCCAGCCAAGG	
	PhtD_F4	AACCAAATGCGCAAATTACC	
	PhtD_R2	TCCTTATTTTCCTGCGAACG	
	PhtD_R3	GGAGGGGTCAAACCTTTCTC	
	PhtD_R4	GGTTGAGCTGGATTTGCATT	

### Allele type determination

DNA sequences were translated into amino acid sequences, and an alignment was performed, which resulted in the identification of different allele types at the protein level. For Ply, these data were compared to previously determined allele types [19], and newly identified allele types were ordered by number. Because alleles of the *phtD* gene have not been reported previously, all allele types of the *phtD* gene were newly assigned in this study, according to their amino acid sequence variations. In addition, *ply* and *phtD* alleles were subtyped according to variations in their nucleotide sequence. For allele typing of both genes, the sequences from the D39 strain were designated as allele 1 and served as reference sequences.

### Phylogenetic analysis

To investigate the evolutionary relationships among the allele types of the *ply* and *phtD* genes in Korea, phylogenetic trees were individually constructed with the full nucleotide sequences of the *ply* and *phtD* genes obtained from the pneumococcal isolates in this study via the neighbor-joining method [20]. All evolutionary trees were drawn using MEGA5 software (http://megasoftware.net/) [21]. Pairwise evolutionary differences were computed using the maximum composite likelihood method in MEGA5. The percentages of replicate trees in which the associated sequences clustered together in the bootstrap test (500 replicates) are reported as the bootstrap values. In addition, based on the phylogenetic tree, a clade type was assigned to the allele groups that shared a main branch of the tree.

### Antigenicity plots

Antigenic patterns of the Ply and PhtD proteins were analyzed to identify the point of greatest local hydrophobicity. This was accomplished by assigning a numerical value (hydrophobicity value) to each amino acid and then taking a moving average of these values along the peptide chain. The point of highest local average hydrophobicity was invariably located in or immediately adjacent to an antigenic determinant [22]. Antigenicity values were calculated, and the relevant diagrams were constructed using the CLC Main Workbench ver. 6.6.5 software (CLC bio, Aarhus, Denmark). Antigenicity plots for Ply and PhtD were compared with each other individually, as well as with plots for the D39 strain, which was used as a reference sequence in a previous study [19]. Antigenicity plots with different amplitudes or numbers of peak hydrophobicity points were defined as having ‘different antigenicity’.

## Results

### Characteristics and serotypes of pneumococcal isolates

All 173 pneumococcal isolates were obtained from sterile body fluid samples from children diagnosed with IPD. The median age of the children was 30.2 (range: 1.2–213.5) months. Twenty-four (13.9%) isolates were collected in 1991–1995, 54 (31.2%) isolates in 1995–2000, 47 (27.2%) isolates in 2001–2005, and 48 (27.7%) isolates in 2006–2011. A total of 27 serotypes were identified; the most common serotypes were 19A (17.3%), followed by 23F (13.9%), 6B (9.8%), and 14 (9.2%).

### Allele types and sequence diversity of the *ply* gene

**Fig 1:**
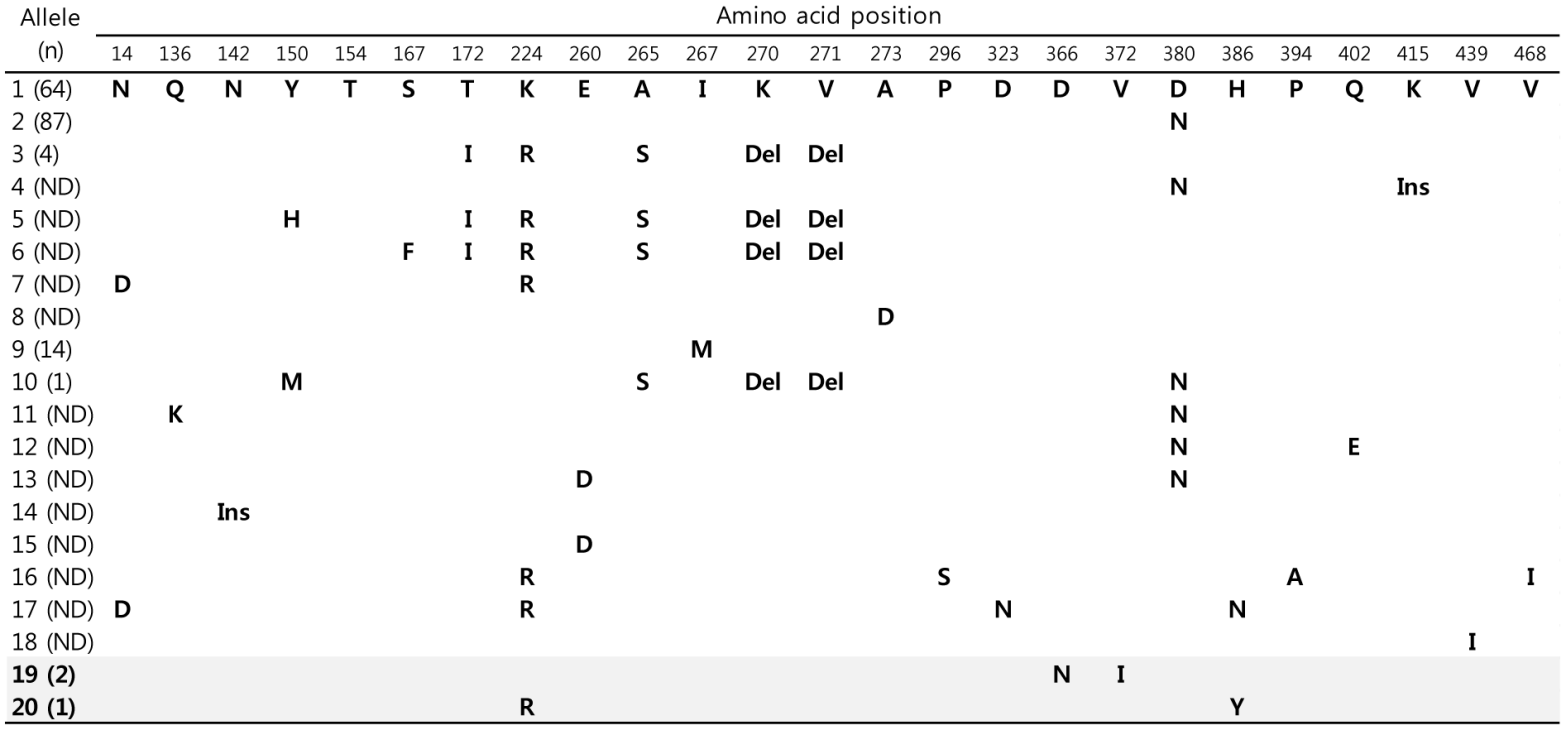
Allele types and corresponding variations in amino acid sequences of pneumolysin (*ply*). The amino acid sequence of allele type 1 (D39 strain) was compared with all other allele types. The novel allele types, 19 and 20, are indicated by shaded rows. Notes: ND, not detected; Del, single amino acid deletion; Ins, 8 (in allele 4) and 291 (in allele 14) amino acid insertions.

**Fig 2:**
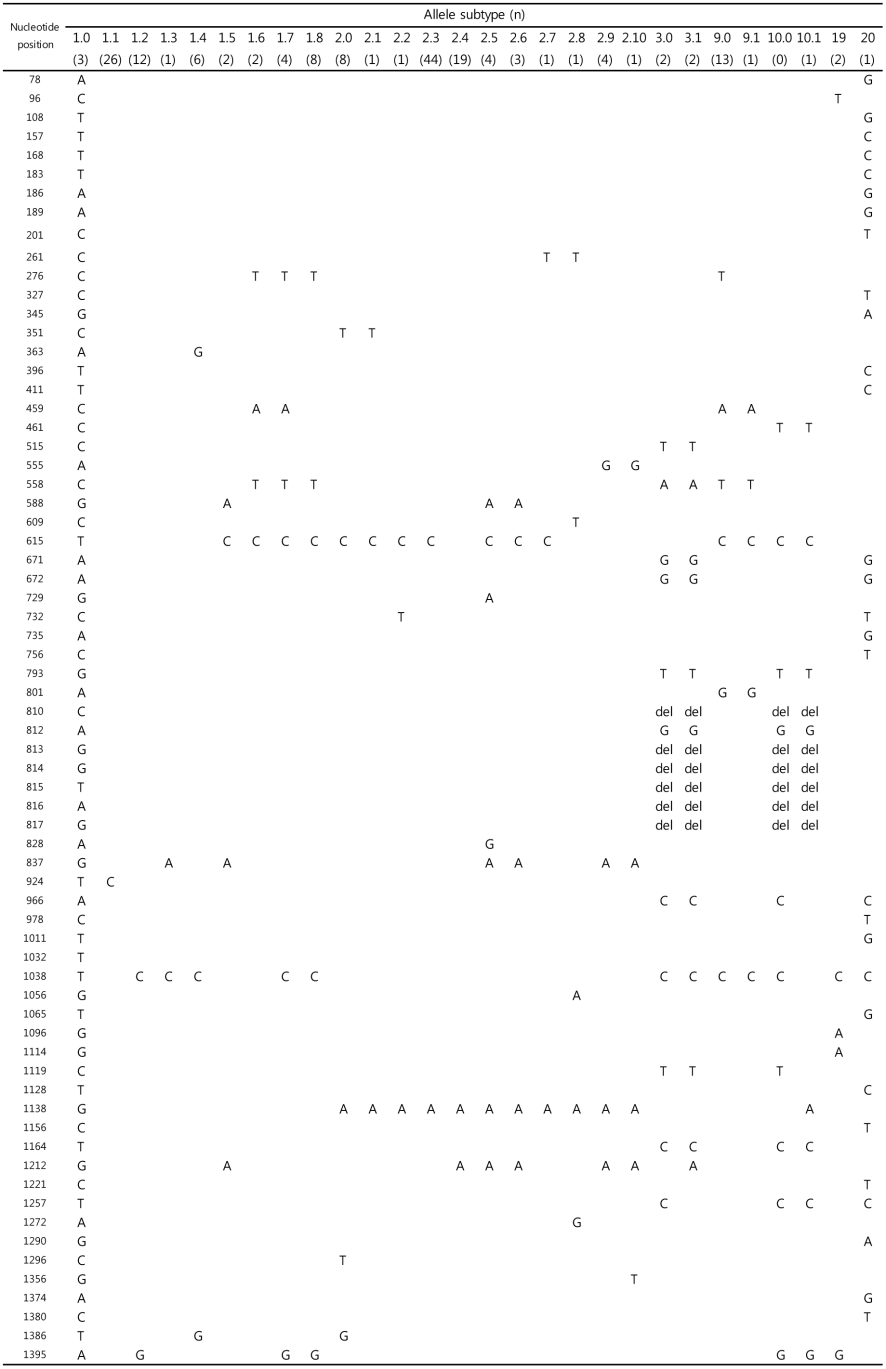
Allele subtypes and corresponding variations of nucleotide sequences of pneumolysin (*Ply*) identified in this study. Nucleotide sequence of allele subtype 1.0 (D39 strain) was compared with that of all other allele types. Note: del, single nucleotide deletion.

Five allele types (1, 2, 3, 9, and 10) were identified from among 18 previously reported types [19]. Two additional allele types, alleles 19 and 20, were newly identified in this study and had two amino acid changes each relative to the reference sequence (D39 strain, allele 1): D366N and V372I in allele 19, and K224R and H386Y in allele 20 (Fig 1). Alleles 1 (n = 64, 37.0%) and 2 (n = 87, 50.3%) were the most commonly observed *ply* alleles, in agreement with a previous study [19]. The numbers of isolates representing the other *ply* allele types were four (2.3%) for allele 3, 14 (8.1%) for allele 9, one (0.6%) for allele 10, two (1.2%) for allele 19, and one (0.6%) for allele 20. The two isolates from which *ply* allele 19 had been obtained were of serotypes 15B and 15C, and the isolate from which *ply* allele 20 had been obtained was of serotype 35B. Subtypes, which are defined as variations in nucleotide sequences that did not change amino acid sequences, were provided in Fig 2.

All *ply* allele types except alleles 4 and 14 were 471 amino acids length. As previously reported [19, 23], *ply* alleles 4 and 14 include insertions (INS) of eight and 291 amino acids, respectively (Fig 1). To facilitate the comparison of the sequence diversity that was common to the *ply* allele types, we excluded these INS from *ply* alleles 4 and 14 from further analyses. When the INS were excluded, the sequences of allele types 4 and 14 were identical to the sequences of allele types 2 and 1, respectively. Greater than 98% amino acid sequence identity (98.3%-100%, pairwise comparisons, data not shown) was found among all *ply* allele types, including the novel allele types 19 and 20. In addition, a nucleotide sequence identity of >95% (96.2%-99.9%) was observed among all known *ply* allele types. When the nucleotide sequences of the 28 *ply* allele subtypes in this study (Fig 2) were compared, sequence identity increased among the subtypes (97.2%-99.9%, pairwise comparisons, data not shown).

### Allele types and sequence diversity of the *phtD* gene

Initially, we identified six allele types from the currently available six sequences of the *phtD* gene from GenBank database (NC_003098, NC_008533, NC_011072, NC_011900, NC_017592, and NC_017593), based on their amino acid sequence variations. In addition, 34 additional allele types were identified in a set of 173 invasive pneumococcal isolates in this study. All *phtD* genes showed one of 12 amino acid lengths, ranged from 833 to 856. Allele number was assigned to each allele type by their amino acid sequence and length, and in identified order, except allele 1 (*phtD* allele 1 was assigned to the D39 reference strain, NC_008533) (Table 2). Allele types in the same amino acid length showed the amino acid sequence identities of >95%, except alleles 39 and 40 (identity of 93.5%, pairwise comparisons). Pairwise comparisons of nucleotide and amino acid sequences for all *phtD* allele types demonstrated 91.4%-99.9% and 90.4%-99.9% identities, respectively.

**Table 2:** Allele types of pneumococcal histidine triad protein D (*phtD*).

Allele	Size (aa)	Number (%) of isolates	Amino acid sequence Identity (%) of isolates*
**1**	854	1 (0.6)	100
**2**	843	0	NA
**3, 4**	842	5 (2.9)	99.41
**5–9**	840	8 (4.6)	96.55–99.17
**10–23**	839	127 (73.4)	97.26–99.88
**24, 25**	833	4 (2.3)	99.64
**26–28**	835	4 (2.3)	98.56–99.88
**29–32**	849	4 (2.3)	95.32–97.65
**33–36**	850	14 (8.1)	98.12–99.53
**37**	856	2 (1.2)	100
**38**	844	1 (0.6)	100
**39, 40**	857	3 (1.7)	93.47

aa, amino acid; NA, not available

*Pairwise comparisons

**Fig 3:**
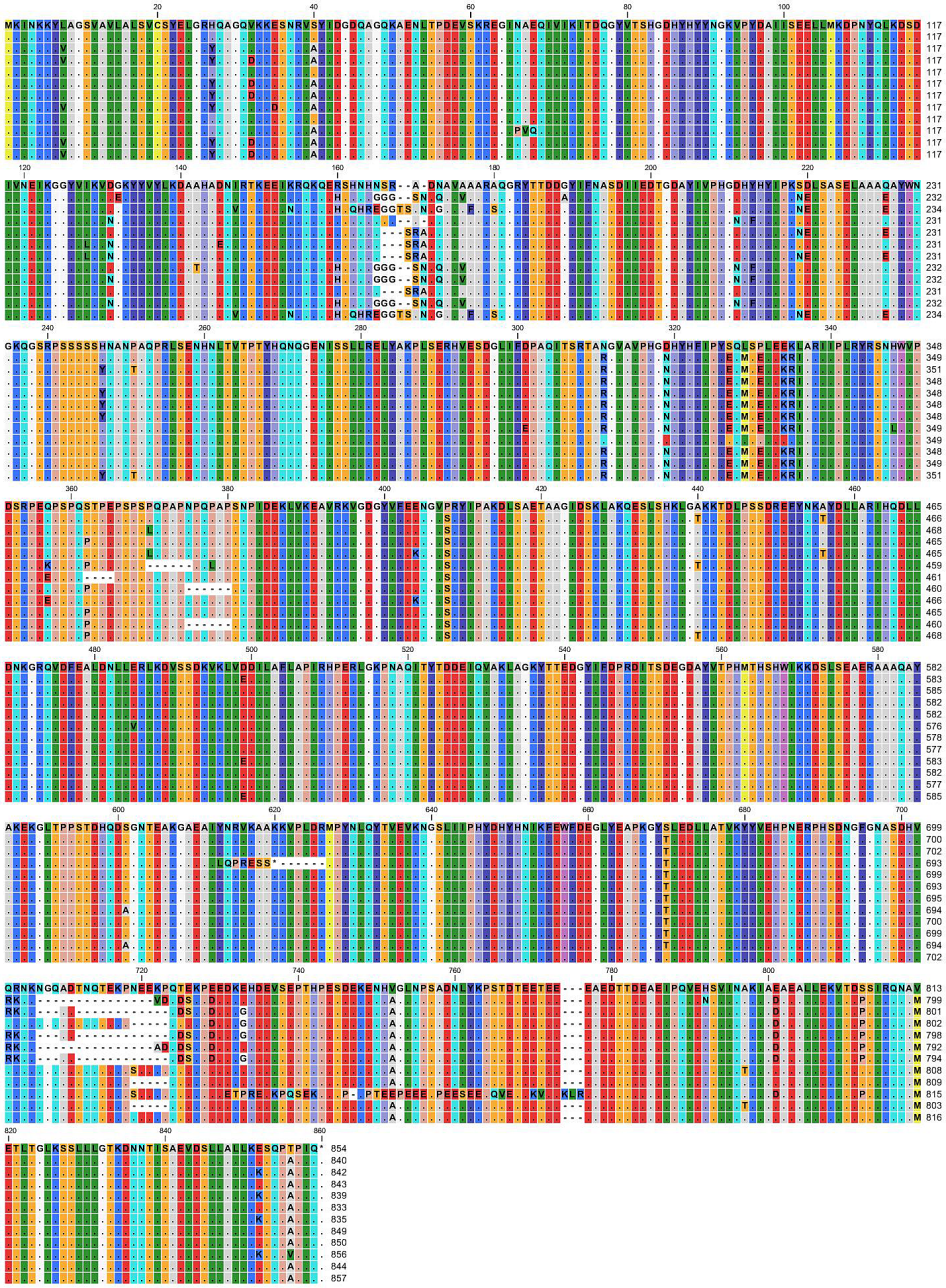
Amino acid sequence alignment for 12 representative allele types of pneumococcal histidine triad protein D (*phtD*). Each one allele type was selected from each length of amino acids of *phtD*. Sequences of allele 1, 2, 3, 5, 10, 24, 26, 29, 33, 37, 38, and 39 were presented in top-down order. Identical residues are shown as dots.

Fourteen allele types (alleles 10–23) and 127 (73.4%) pneumococcal isolates showed an amino acid length of 839 (Table 2). *phtD* allele 2, assigned from a GenBank strain (NC_011072), was not identified in this study. Among 39 allele types of *phtD* gene, only the allele types 3 and 10 were divided to two subtypes according to their nucleotide sequence variation. Only one nucleotide sequence each differed between the two subtypes both in *phtD* alleles 3 (C2802T) and 10 (G1296T). Amino acid sequence alignments for 12 representative allele types of the *phtD*, which were each one allele type in different length of amino acid, are presented in Fig 3.

### Phylogenetic analysis of the *ply* and *phtD* genes

Phylogenetic trees were constructed based on the nucleotide sequences of the entire *ply* and *phtD* genes, respectively. The nucleotide sequences of all allele subtypes of the *ply* gene from pneumococcal isolates in this study were aligned for neighbor-joining analysis. As the allele types of *phtD* gene in the same amino acid lengths have a large degree of homogeneous nucleotide sequences, we analyzed 12 representative allele types of the *phtD* gene instead of all 39 allele types.

**Fig 4:**
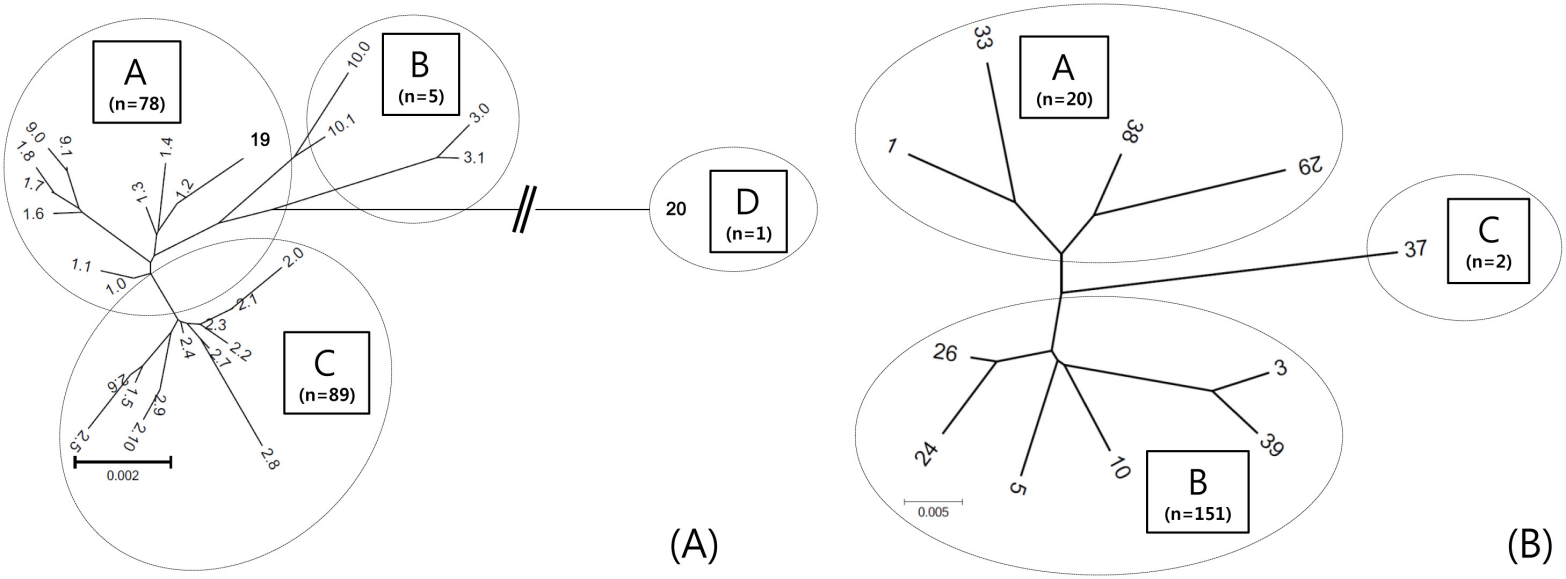
Phylogenetic analysis of all pneumolysin (A) and pneumococcal histidine triad protein D (B) alleles. Phylogenetic and molecular evolutionary analyses were performed with the MEGA5 program using the neighbor-joining method. The numbers at the ends of the tree and the characters (A, B, C, and D) in the square boxes indicate allele and clade types for *ply* or *phtD*, respectively. The bar at the bottom represents genetic distance. Bootstrap support based on 500 replicates were 41% for A and B, 59% for A and C, and 91% for B and D in Fig 4A and. 86% for A and B and 49% for A and C in Fig 4B.

For the *ply* gene, one of four clades (A, B, C, and D) was assigned to each allele subtype according to the main branch from which it was derived (Fig 4A). *Ply* allele type 19 clustered with allele 1 (clade A, defined in this study), and allele type 20 was placed in the furthest diverged clade D. For the *phtD* gene, we classified allele types into three clades, specifically A, B, and C (Fig 4B).

### Antigenicity plots of the *ply* and *phtD* genes

**Fig 5:**
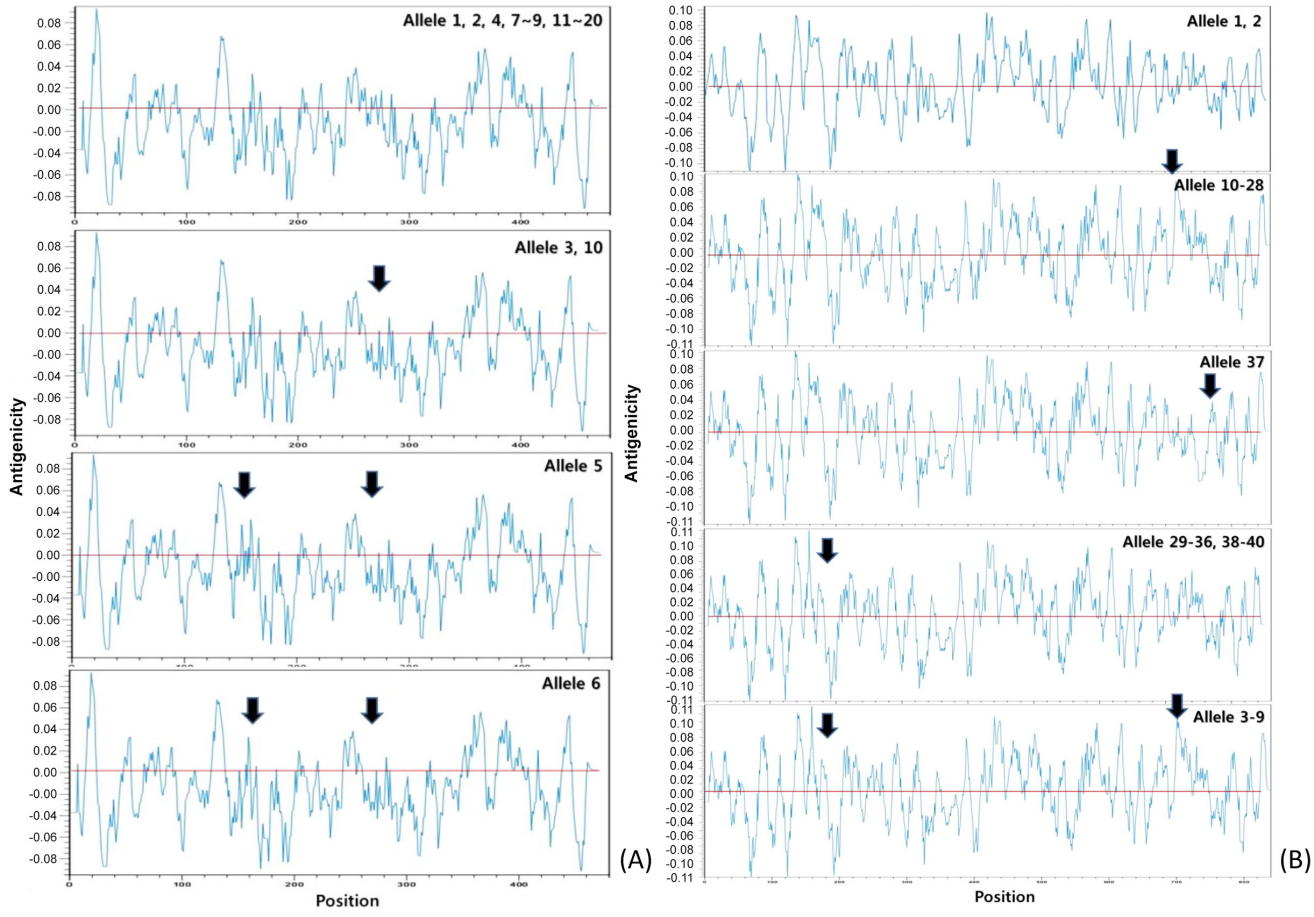
Comparison of the antigenicity plots of pneumolysin (A) and pneumococcal histidine triad protein D (B) alleles. The vertical axis represents antigenicity values. Closed black arrows indicate the residues with antigenicity values different from that of the D39 strain (allele 1) reference sequence. The insertion sequences in alleles 4 and 14 of pneumolysin were excluded in this comparison for simplicity.

The majority of *ply* allele types, including the novel alleles 19 and 20, exhibited the same antigenicity plots. Exceptions to this were alleles 3, 5, 6, and 10, which included only 1–2 positions that differed from reference allele 1, in positions spanning amino acids 150–170 (YEKITAHSMEQ; Y150H in allele 5 and S167F in allele 6) and 270–280 (VKVAPQTEWKQ; del[270K, 271V] in alleles 3, 5, 6, and 10; Figs 1 and 5A). Among our isolates, only five (2.9%; four for allele 3 and one for allele 10) isolates exhibited different antigenicity plots.

Similarly, the *phtD* alleles were classified into five antigenicity patterns. Isolates of *phtD* alleles 10–28 (n = 135, 78.0%) exhibited the same antigenicity pattern, while the other isolates (n = 31, 17.9%), except those of alleles 3–9 (n = 7, 4.0%), showed a similar antigenicity pattern with a minor difference at a single position. Three positions differed from each other within amino acids 160–170 (RSHNHNSRADN **→** HSQHREGGTSANDG in alleles 3, 4, 39, and 40 and HSHNHGGGSNDQ in alleles 5–9, 29–36, and 38), 700–720 (QRNKNGQADTNQTEKPNEEKP **→** RKNKVD in alleles 5–9 and RKNKAD in alleles 3, 4, and 10–28), and 770 (addition of the three amino acid sequence, KLR in allele 37; Figs 3 and 5B).

## Discussion

In this work, we characterized the sequence variations and antigenicities of the *ply* and *phtD* genes from 173 pneumococci that were isolated from invasive diseases in children between 1991 and 2011. Overall genetic identity with respect to the nucleotide sequences of *ply* and *phtD* in our isolates was greater than 97% and 91%, respectively, and an exclusively homogeneous pattern was seen in the antigenicity plots for both genes. We report the presence of seven *ply* alleles from a collection of 173 isolates, two of which are novel allelic variants. Moreover, we believe this to be the first study examining the allelic distribution of the *phtD* gene among clinical isolates of *S*. *pneumoniae*.

Ply is produced by all known clinical isolates of *S*. *pneumoniae*, irrespective of serotype and genotype. The amino acid sequence of Ply is thought to be highly conserved over time and geographic distance [23]. However, others have reported the existence of at least 18 different Ply variants at the protein level, especially in carriage isolates [19]. The genes of Ply were detected in all invasive pneumococcal isolates in this study and exhibited very stable sequences over 21 years, although some allelic variations were present. When amino acid insertions were excluded from alleles 4 and 14 for comparison, all known allele types, including the novel types 19 and 20, also demonstrated stable antigenic properties. Moreover, insertions in the alleles 4 and 14 did not have a large impact on the antigenicity plot and pneumococcal strains with *ply* alleles 4 and 14 were not identified in this study. Only the two amino acid substitutions at residues 150 and 167 and two amino acid deletions at residues 270 and 271 produced subtle changes in antigenicity values at these locations. Ply residues 150–167 and 270–271 are included in Ply domains 1 and 3, respectively. Among the four domains of Ply, domains 1 and 3 are expected to have important roles in inducing conformational changes in the Ply oligomer, resulting in membrane deformation of a target cell [24]. Further studies are needed to determine whether minor sequence changes in domains 1 and 3 of Ply, as observed in the present study, can influence pneumococcal virulence.

There is a high degree of protein sequence conservation among PhtD proteins from diverse *S*. *pneumoniae* serotypes, and a recent study showed that PhtD is present in 100% of known strains [25]. The genes of PhtD were also detected in all 173 invasive isolates in the current study. Seventy-three percent of isolates had a size of 839 amino acids, which was designated as alleles 10–23. Although amino acid sequence and allelic variations in *phtD* genes were observed, nucleotide as well as amino acid sequences were not very diverse among the different allele types. In the phylogenetic analysis, clade B contained *PhtD* allele types 3, 5, 10, 24, 26, and 39, which comprised a total of 151 (87.3%) isolates in this study. Moreover, the antigenicity patterns of PhtD were much more homogeneous than expected based on genetic identity. All three regions differed primarily in terms of their antigenicity patterns, and residues 160–170, 700–720, and 770, in which variations occurred, were included in the regions that do not produce functional domains [26]. Whether these PhtD sequence variations can affect the virulence of pneumococci should be evaluated further.

This study has some limitations. In this study, among all the known 18 alleles of *ply*, only 7 allele were identified. Although this can represent sequence homogeneity of invasive pneumococcal isolates in Korea, this should be confirmed by further researches with larger sample sizes and more diverse geographical sampling. This study explored the diversity and antigenicity of the *ply* and *phtD* of the invasive isolates only. Further studies should be extended to determine the diversity of the pneumococcal carriage strains.

Sequence conservation is a necessary requirement for a universal pneumococcal vaccine. Few studies have investigated the sequence conservation of *ply* and *phtD* among invasive pneumococcal isolates from children. To investigate the extent of conservation of the *ply* and *phtD* genes, we sequenced *ply* and *phtD* from 173 pneumococcal isolates and compared the sequences to each other and to sequences in the GenBank database. In this study, the genetic and antigenic diversities of Ply and PhtD were very limited. The apparent ubiquity of Ply and PhtD along with their presumed lack of antigenic variability could make them attractive candidates for serotype-independent pneumococcal vaccines. It is also thought that the minor antigenic variations in Ply and/or PhtD in some strains could exert some influence on their virulence, a concept that should be explored.
